# Cardiac Arrhythmia classification based on 3D recurrence plot analysis and deep learning

**DOI:** 10.3389/fphys.2022.956320

**Published:** 2022-07-22

**Authors:** Hua Zhang, Chengyu Liu, Fangfang Tang, Mingyan Li, Dongxia Zhang, Ling Xia, Nan Zhao, Sheng Li, Stuart Crozier, Wenlong Xu, Feng Liu

**Affiliations:** ^1^ School of Information Technology and Electrical Engineering, University of Queensland, Brisbane, QLD, Australia; ^2^ School of Instrument Science and Engineering, Southeast University, Nanjing, China; ^3^ Zhejiang Provincial Centre for Disease Control and Prevention CN, Hangzhou, China; ^4^ Department of Biomedical Engineering, Zhejiang University, Hangzhou, China; ^5^ The College of Science, Xijing University, Xi’an, China; ^6^ Department of Biomedical Engineering, China Jiliang University, Hangzhou, China

**Keywords:** cardiac arrhythmia classification, electrocardiogram, recurrence plot, vectorcardiography, deep learning

## Abstract

Artificial intelligence (AI) aided cardiac arrhythmia (CA) classification has been an emerging research topic. Existing AI-based classification methods commonly analyze electrocardiogram (ECG) signals in lower dimensions, using one-dimensional (1D) temporal signals or two-dimensional (2D) images, which, however, may have limited capability in characterizing lead-wise spatiotemporal correlations, which are critical to the classification accuracy. In addition, existing methods mostly assume that the ECG data are linear temporal signals. This assumption may not accurately represent the nonlinear, nonstationary nature of the cardiac electrophysiological process. In this work, we have developed a three-dimensional (3D) recurrence plot (RP)-based deep learning algorithm to explore the nonlinear recurrent features of ECG and Vectorcardiography (VCG) signals, aiming to improve the arrhythmia classification performance. The 3D ECG/VCG images are generated from standard 12 lead ECG and 3 lead VCG signals for neural network training, validation, and testing. The superiority and effectiveness of the proposed method are validated by various experiments. Based on the PTB-XL dataset, the proposed method achieved an average F1 score of 0.9254 for the 3D ECG-based case and 0.9350 for the 3D VCG-based case. In contrast, recently published 1D and 2D ECG-based CA classification methods yielded lower average F1 scores of 0.843 and 0.9015, respectively. Thus, the improved performance and visual interpretability make the proposed 3D RP-based method appealing for practical CA classification.

## Introduction

Cardiovascular Diseases (CVD) are a leading cause of death globally (Sahin and Ilgun, 2020; Amini et al., 2021). Cardiac arrhythmia is a common CVD associated with disorganized electrical activities of the heart. Several main types of arrhythmias include Atrial Fibrillation (AF), First-degree Atrioventricular Block (I-AVB), Bundle Branch Block (BBB), and so on. Some arrhythmias can significantly impact the patient’s health, such as AF, which can pose a significant risk for stroke (Ye et al., 2012; Siontis et al., 2021), while others are common and relatively harmless. It is essential to classify the risk types as early as possible to manage and treat arrhythmia-associated heart diseases. Manual interpretation of the electrocardiogram (ECG) is an effective and non-invasive way for arrhythmia classification and diagnosis. Traditional ECG-based arrhythmia diagnostics require considerable expertise; recently, computer-aided ECG diagnosis for arrhythmia based on machine learning and deep learning has become an active research area (Siontis et al., 2021).

In traditional machine learning methods, a set of timing and morphology features of ECG signals were extracted and discriminated by learning-based classifiers (De Chazal et al., 2004; De Chazal and Reilly, 2006; Ince et al., 2009; Ye et al., 2012). (Asl et al., 2008) extracted the R-R interval features from the raw ECG signals and then employed a support vector machine classifier to discriminate six types of arrhythmias. (Llamedo and Martinez, 2011). used features extracted from the R-R series and computed from different scales of the wavelet transform for arrhythmia classification by a linear classifier. In general, these methods heavily rely on in-depth domain knowledge. Furthermore, the extracted hand-crafted features from the ECG signals can vary among patients, making it challenging to maintain both the accuracy and generalization of arrhythmia classification.

Deep learning networks have been widely utilized to perform automated feature extraction based on raw or low-level processed ECG data and achieve end-to-end arrhythmia classification (Siontis et al., 2021). Existing studies have demonstrated the effectiveness of ECG feature detection in predicting arrhythmia. Most of them focus on features of ECG signals, including one-dimensional (1D) time-domain features (e.g., directly taking ECG series as input signals), frequency and time-frequency domain features (e.g., Fourier transform, wavelets transform), and ECG morphology-based image features (e.g., using 2D grayscale images). For the 1D time-domain features, Hannun et al. developed a deep neural network to classify 12 types of arrhythmias based on single-lead ECG time signals. The prediction performance exceeds that of the average cardiologist (Hannun et al., 2019). Some other studies combined a recurrent neural network, such as the long-short term memory (LSTM), with a convolution neural network (CNN) to capture the historical information of the ECG (He et al., 2019; Chen et al., 2020; Yao et al., 2020; Rahul and Sharma, 2022b). For the frequency and time-frequency domain features of ECG, researchers attempted to convert the 1D ECG signals into 2D images to predict different types of CA. Huang et al. transferred the 1D ECG time signals to 2D time-frequency spectrograms, then transformed the arrhythmia identify task into an image classification task based on a 2D CNN(Huang et al., 2019). Jagdeep Rahul et al. transformed the 1D ECG into 2D time-frequency representation as the input, then fed it into the Bi-directional LSTM model for AF prediction (Rahul and Sharma, 2022a). (Li et al., 2019) developed an approach based on three types of wavelets transform and the 2D CNN to detect Ventricular ectopic beat in the image domain. For the ECG morphology-based image features, 1D ECG signals were converted into 2D grayscale images and then fed into 2D CNN to classify different arrhythmia types (Izci et al., 2019). Most of these classification methods have been designed for detecting linear, time-frequency features of ECG signals. However, the human heart is a complex, dynamic system (Zbilut et al., 2002), generating ECG signals naturally nonstationary and nonlinear (Acharya et al., 2011). Therefore, the methods mentioned above might be incapable of fully characterizing the dynamical nature of the ECG signals.

To study nonlinear dynamic spatial features of the cardiac system for arrhythmia classification, the recurrence plot (RP) technique has been used to discover the recurrence pattern buried in the time series of ECG signals and then successfully applied to the detection of ventricular fibrillation, as well as the prediction of premature atrial complex, premature ventricular complex, and AF (Mathunjwa et al., 2021). In our recent work (Zhang et al., 2021), we successfully utilized the 2D RPs to distinguish various arrhythmias, leading to better solutions than linear approaches.

This work aims to develop further the RP technique into a 3D framework for improved arrhythmia classification. In our recent study (Zhang et al., 2021), the 2DRP images offer a unique feature detection mechanism for arrhythmia classification compared with conventional approaches. However, those 2DRP maps are directly fed into the neural network in a decoupled manner, without sorting and directly analyzing shared features and nonlinear alterations between these 2D images in the training process. The new 3DRP maps-based deep learning training process allows the neural network to extract the correlation between the ECG leads, thus explicitly offering more comprehensive recurrence features in the phase space that help identify the uniqueness of each type. In implementing 3D RP-based arrhythmia classification, we compared two 3D transforms, namely the ECG-based and VCG-based methods.

The contributions of this work include: 1) this is the first study using the RP technique for mapping 12 lead ECG signals to 3DRP texture images and performing deep learning-based arrhythmia classification; 2) the 3 lead VCG was introduced into the RP method to efficiently extract the nonlinear features of the ECG signals for optimized arrhythmia prediction; 3) the proposed 3D Inception Resnet model was used to extract the spatial pattern features and textural alternations from the 3D RP images.

The rest of the paper is organized as follows: the approach and the network architecture are described in *Methodology Section*, the experiments are detailed in *Experiment Section*, the discussion on results is provided in *Discussion Section*, and conclusions are drawn in *Conclusion Section*.

## Methodology

In this section, the arrhythmia classification task is treated as a 3D ECG image classification problem using the proposed 3D RP technique and the 3D Inception Resnet model.

### Recurrence plot

Recurrence is one of the fundamental properties of a dynamic system, such as the electrical signals generated by the human heart, and is difficult to detect in serial time-domain signals (Marwan et al., 2007; Debayle et al., 2018). The Recurrence Plot (RP) approach was proposed to explore the phase space trajectory in a higher-dimensional space and to show the recurrent behaviors of the time series (Eckmann et al., 1987; Eckmann et al., 1995).

An RP can be formulated as follows:

$$R_{i,j}\;=\theta \left(\varepsilon -\left\|x_{i}-x_{j}\right\|\right),\;i,j=1,....,N$$ (1)

where N is the number of time series $x_{i}$ , ε is a predefined distance, ‖·‖ is an L2 norm, and θ(.) is the Heaviside function.

θ(.) is defined as:

$$\theta \left(Z\right)=\{\begin{array}{cc} 0, & \mathrm{if}\;\mathrm{z<0}\\ 1, & \mathrm{otherwise} \end{array}$$ (2)

Eq. 1 is considered binary because of the predefined distance. For this study, an un-threshold approach (Faria et al., 2016) was applied to obtain more information contained in the RP images. Specifically, The R-matrix can be defined as:

$$R_{\mathrm{i,j}}=\left\|x_{i}-x_{j}\right\|,\;\mathrm{i,j= 1,}....\mathrm{,N}$$ (3)

### Vectorcardiography

To reduce the data size for neural network training, we consider converting the standard 12-lead ECG signals into VCG signals for deep learning-based arrhythmia classification. VCG was introduced by (Frank, 1956). Since the human body is a 3D structure, the basic idea of VCG is to construct three orthogonal leads containing all the electric information of the human heart. The three leads are represented by the right-left axis (Vx), head-to-feet axis (Vy), and front-back (anteroposterior) axis (Vz). Based on the standard 12-lead system, the following expressions are used to calculate Frank’s leads Vx, Vy, and Vz (Daniel et al., 2007).

Vx=−(−0.172V1−0.074V2+0.122V3+0.231V4+0.239V5+0.194V6+0.156DΙ−0.010DΙΙ) (4)

Vy=(0.057 V1−0.019 V2−0.106 V3−0.022 V4+0.041 V5 +0.048 V6 −227 DΙ +0.887 DΙΙ) (5)

**FIGURE 1:**
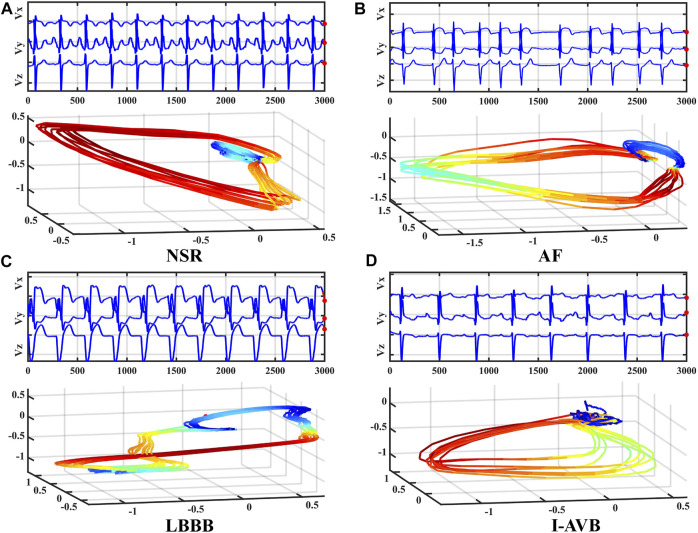
Frank’s three leads signal Vx, Vy, and Vz of four types of VCG waveforms (top) and corresponding 3D dynamic feature plots (bottom).

Vz=−(−0.229 V1−0.310 V2−0.246 V3−0.063 V4+0.055 V5+0.108 V6+0.022 DΙ+0.102 DΙΙ) (6)

where DΙ and DΙΙ are the leads I and II, and V1-V6 are the chest leads (V1, V2, V3, V4, V5, V6) of 12-lead ECG. Even though the converted VCG is not widely used as the ECG, it records essential features of cardiac electrical excitation changes over time. It has been shown that over 90% of ECG energy can be reserved by the 3-lead VCG (Hasan et al., 2012). As illustrated in Figure 1, VCG signals reflect the heart’s electrical activities in both spatial and temporal domains through three orthogonal planes of the body (Yang et al., 2012). The dynamic differences between the VCG signals can thus be used for arrhythmia classification.

### 3DRP Inception Resnet architecture

**FIGURE 2:**
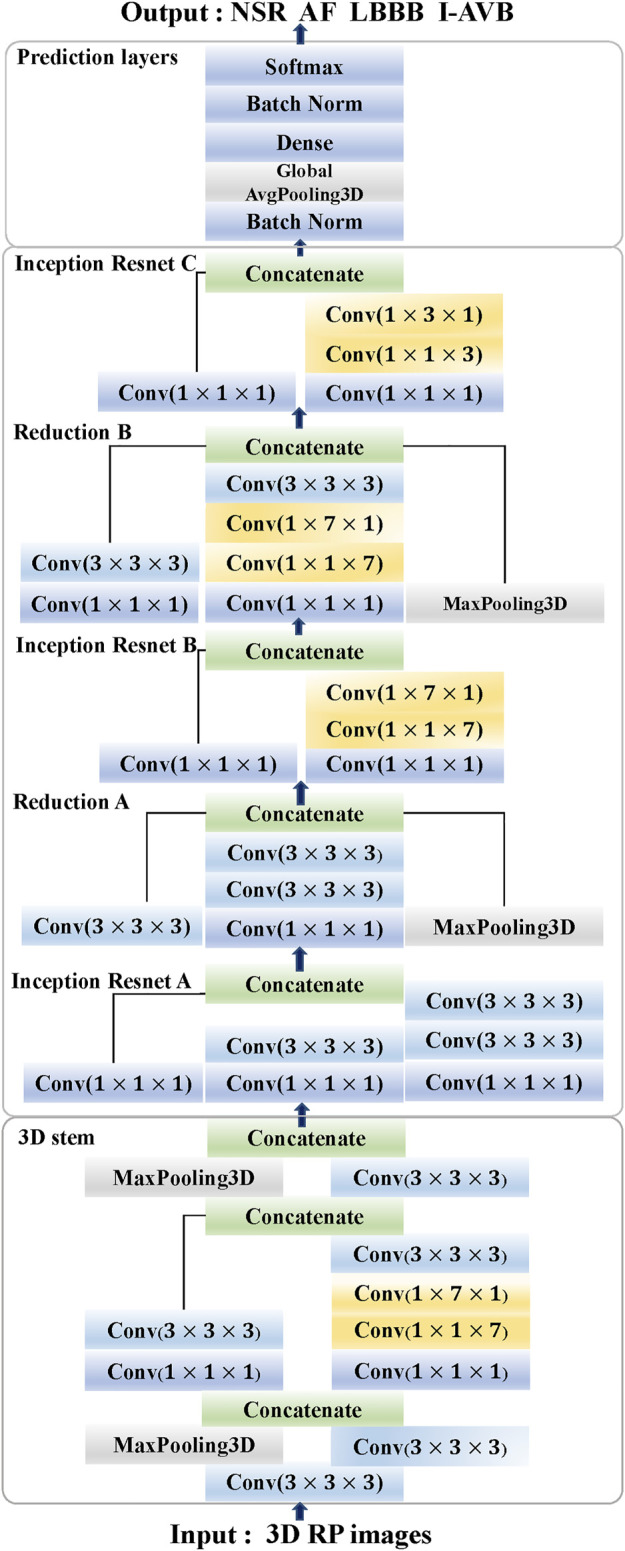
The architecture of 3DRP Inception ResNet (Stem, Inception ResNet models **A-C**, Reduction models **A and B**, and Prediction layers).

The proposed 3DRP Inception Resnet network was designed based on the Inception-ResNet-v2 (Szegedy et al., 2017). In this study, we expanded the network from 2D to 3D and improved the Inception Resnet models, as shown in Figure 2. It contains the 3D Stem, the 3D Inception Resnet models, and the 3D prediction part. In the first part, the 3D Stem model contains deep convolutional layers with 1 × 1 × 1, 3 × 3 × 3, 1 × 1 × 7, 1 × 7 × 1 convolutions, and two max-pooling layers, which are used to pre-process the original data before entering the 3D Inception Resnet blocks. The following part has the 3D Inception Resnet models, including 3D Inception Resnet A and 3D Reduction A with 1 × 1 × 1, 3 × 3 × 3 convolution layers; 3D Inception ResNet B and 3D Reduction B with 1 × 1 × 1, 3 × 3 × 3 convolutions, and 1 × 1 × 7, 1 × 7 × 1 asymmetric filter; 3D Inception ResNet C with 1 × 1 × 1 convolutions, 1 × 1 × 3 and 1 × 3 × 1 asymmetric filter. The network enhances the diversity of the filter patterns by asymmetric convolution splitting. The last part is the prediction layer, including 3D Global Average pooling and SoftMax layers.

## Experiment

### Experimental setup

#### ECG database

The dataset Physikalisch-Technische Bundesanstalt (PTB-XL) (Wagner et al., 2020) from the PhysioNet/Computing in Cardiology Challenge 2020 (Alday et al., 2021) was used in this study. It was illustrated in Table 1, which is composed of four typical CA types labelled as Sinus rhythm (NSR), Atrial fibrillation (AF), 1st degree AV block (I-AVB), and Left bundle branch block (LBBB). Each data contains 12-lead ECG recordings with a sampling frequency of 500 Hz and a mean duration of 10 s. NSR is a normal heart rhythm; AF is related to irregular heart rate, which can lead to an increase in the risk of strokes; I-AVB is a condition of abnormally slow conduction through the atrioventricular node; LBBB is a condition of delay or blockage of electrical impulses along the left side pathway of the heart ventricles bottom.

**TABLE 1:** Data profile for the ECG dataset.

CA types	Number of data	Single-label data	Experiment segments	80%		20%
				Training	Validation	Test
NSR	18092	16801	1200	768	192	240
AF	1514	1396	1200	768	192	240
LBBB	536	370	1110	710	178	222
I-AVB	797	689	1378	883	221	274

#### Data splitting and augmentation

The data from the PTB-XL database were pre-processed and augmented. The raw ECG data were downsampled to 200 Hz. In the first phase, the data with multi-labels were removed initially because we mainly focused on single-labelled arrhythmia classification in this study. After then, the number is 16801 for NSR, 1396 for AF, 370 for LBBB, and 689 for I-AVB. The number of four types of arrhythmias is unbalanced, which brings challenges to the arrhythmia classification. In the second phase, we randomly picked up 1200 data on Sinus rhythm and 1200 data on AF. Four in five of each type of data were used as the training and validation dataset, and one in five was used as the test dataset. Thus, the training set is independent of the testing set, usually called inter-patient classification (Huang et al., 2014). In the third phase, to balance the data in different types, the data was split into a set of 5 s (1000 samples) recordings. Regarding the NSR and AF, we picked up the data from 1st to 1000th; for the LBBB, the data was split into 1st to 1000th, 500th to 1500th, and 1001th to 2000th three segments; for the I-AVB, the data were split into 1st to 1000th and 1001th to 2000th two segments. Thus, 1200 segments of NSR, 1200 of AF, 1100 of LBBB, and 1378 of I-AVB were obtained for experiments. The details of the training, validation and test datasets are provided in Table 1.

#### Classification computing environment

The experiments were performed on the University of Queensland’s computer cluster with 4 × Nvidia Volta V100 SXM2 connected GPUs per node. Each node contains 5,120 CUDA cores, 640 TensorFlow hardware cores, and 32 GB of HBM2 class memory. This model was implemented using the TensorFlow 3.6 and Karas DL framework.

#### Performance of experiments

To assess the effectiveness of the proposed method, several parameters, including Precision, Recall, and F1-score, are defined as follows, respectively.

$$\mathrm{Precision}=\frac{\mathrm{TP}}{\mathrm{TP}+\mathrm{FP}}$$ (7)

$$\mathrm{Recall}=\frac{\mathrm{TP}}{\mathrm{TP}+\mathrm{FN}}$$ (8)

$$F1=\frac{2\left(\mathrm{Precision}\times \mathrm{Recall}\right)}{\mathrm{Precision}+\mathrm{Recall}}$$ (9)

where TP is the number of true positives data; FP is the number of false positives data; FN is the number of false-negative data. Here, Precision is the fraction of all predicted data that are real labeled data, whereas Recall is the fraction of all real labeled data that are successfully detected. The average F1-score among classes is computed to evaluate the final performance of the model. Arrhythmia classification experiments based on ECG and VCG 3DRP methods.

### Arrhythmia classification experiments based on ECG and VCG 3DRP methods

#### Experimental design

**FIGURE 3:**
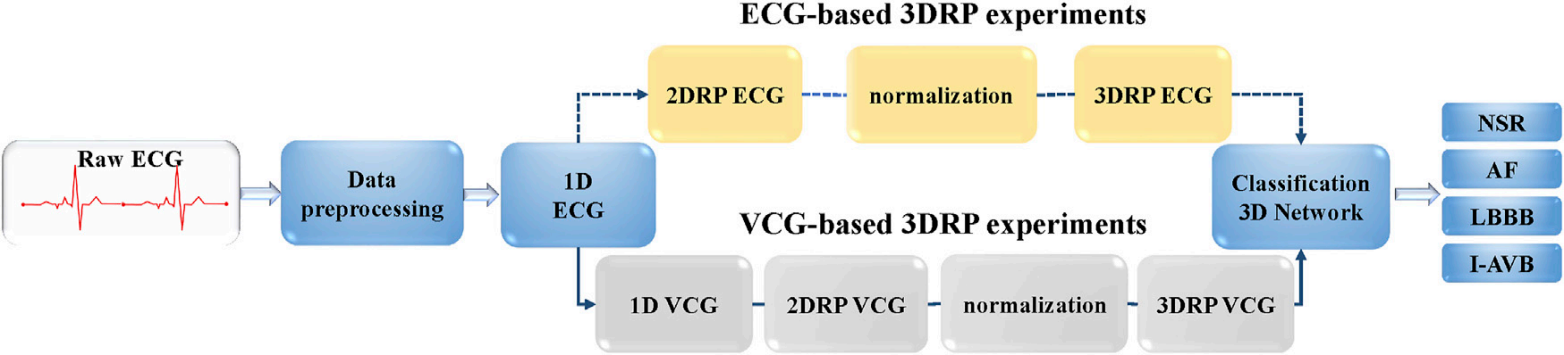
The flow chart of CA classification experiments.

This study aims to investigate the ability of 3DRP to identify pattern differences between various arrhythmia groups. As shown in Figure 3, firstly, the raw ECG data were pre-processed via two steps. In step one, the multi-label data were filtered and divided into four in five for training and validation and one in five for testing. In step two, the data were resampled to 200 Hz and then was augmented by splitting into 5-s recordings to balance the four types of arrhythmias (see section A: Data splitting and augmentation). Then, to explore nonlinear and channel correlation features from the 3D RP images for the arrhythmia classification, ECG-based and VCG-based 3DRP experiments were designed.

**FIGURE 4:**
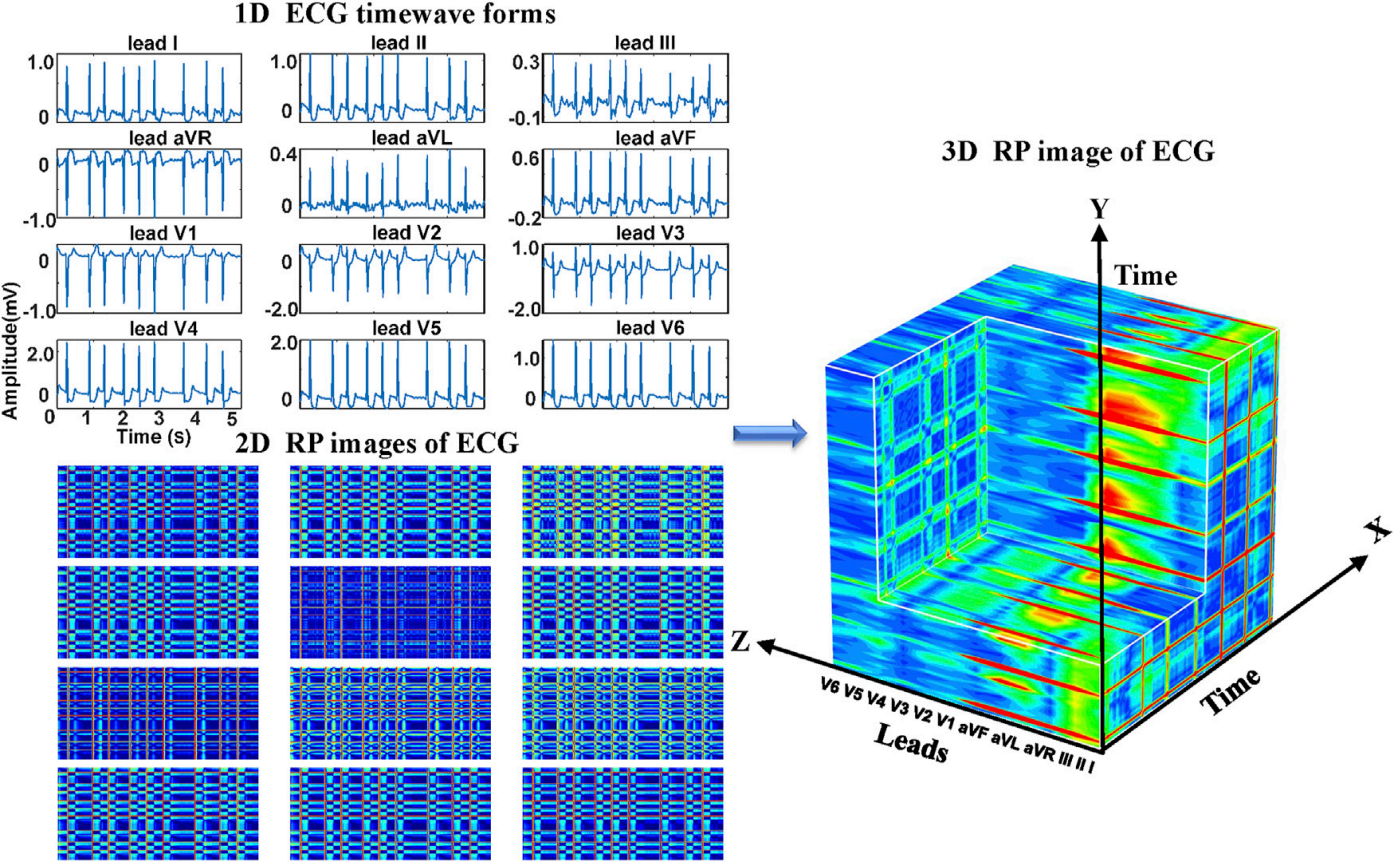
The 3DRP image reconstructed based on ECG.

**FIGURE 5:**
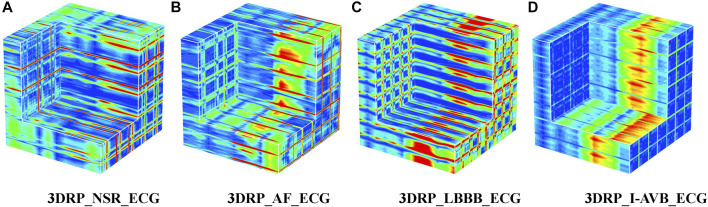
The 3DRP images of NSR/AF/LBBB/I-AVB based on ECG.

Regarding the ECG-based experiments, the 12-lead ECG signals were transformed into 2DRP images and stacked together to form 3D images, as illustrated in Figure 4. The method of converting 1D ECG signals into the corresponding 2DRP images is reported in our previous work (Zhang et al., 2021). Then we applied with (min-max and z-score normalization) and without normalization to pre-process the 2D RPs, respectively, which are defined as follows.

$$RP_{\min-\max}=\frac{\mathrm{RP}-\min}{\max-\min}$$ (10)

$$RP_{z-\mathrm{score}}=\frac{\mathrm{RP}-\bar{\mu }}{\sigma }$$ (11)

where RP is the original data, and min and max are the minima and maximum values of the data. $\bar{\mu }$ and σ refer to its mean value and standard deviation. After normalization, these 12 leads images were placed with the lead-index order of limb leads (lead I, II, III, aVR, aVL, aVF) followed by the chest leads (V1, V2, V3, V4, V5, V6) to form as a 3DRP image. In our previous work (Zhang et al., 2021), we used those 2D RP plots (see Figure 4) to train the network and detect 2D RP features for classification. The relationship between the leads is implicitly investigated by the network, which is essential to explore but less obvious to learn from the 2D textures. In contrast, by setting the 3D RP images as input signals, one can more explicitly discover the inherent signal correlations between the leads in addition to the 2D features within each lead, thus providing higher dimensional, visually interpretable information for prediction. As depicted in Figure 5, significantly different RP patterns can be observed in those 3DRP images obtained from 12-lead ECG data of different arrhythmia types. The texture variations occur within the RP plots and between the leads, which the 3D neural network can easily learn and discriminate the arrhythmia types.

**FIGURE 6:**
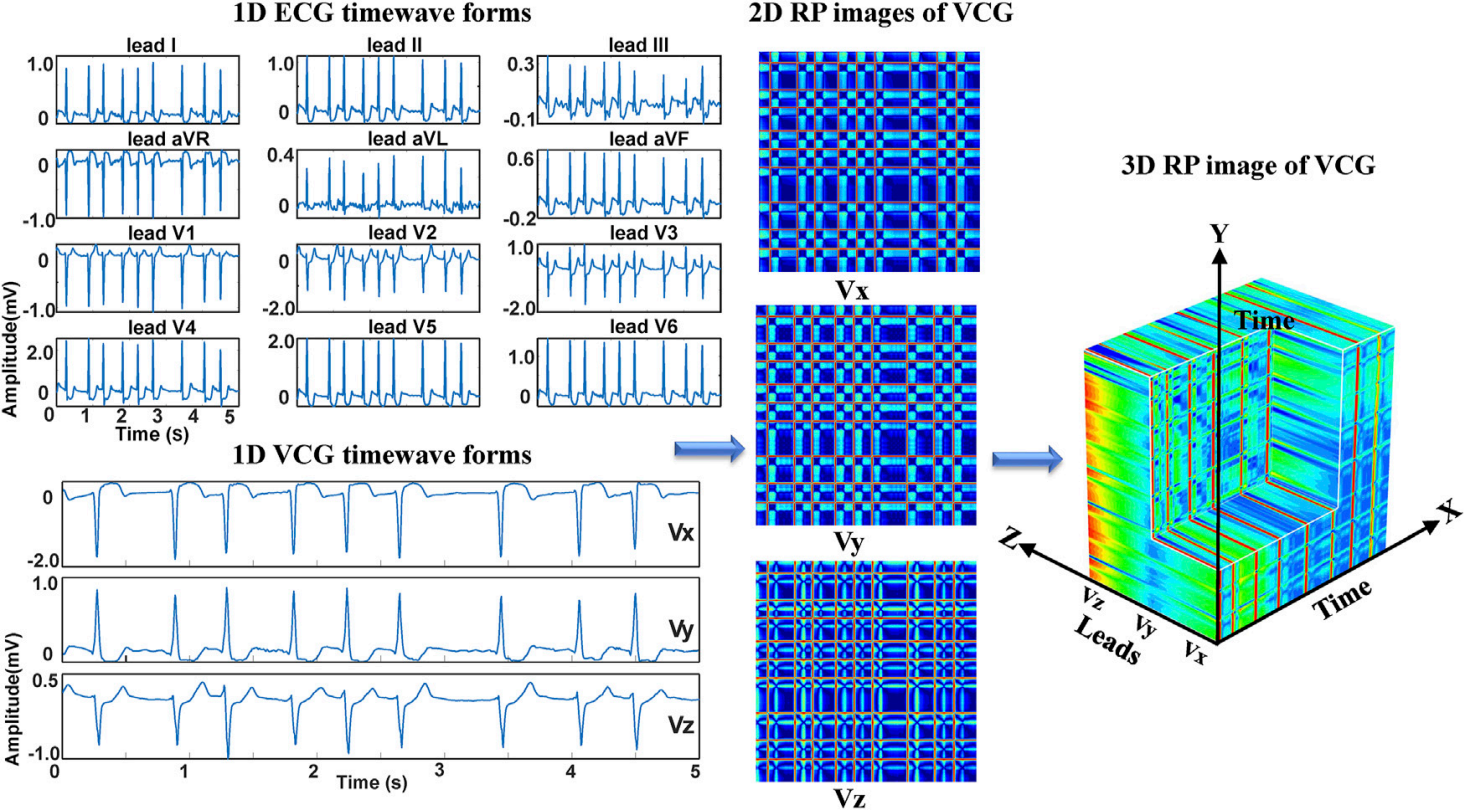
The 3DRP image reconstructed based on VCG.

**FIGURE 7:**
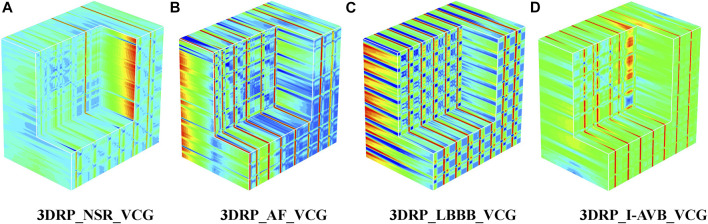
The 3DRP images of NSR/AF/LBBB/I-AVB based on VCG.

Regarding the VCG-based experiments, we investigated VCG-based arrhythmia classification. As shown in Figure 6, we first transformed the pre-processed 1D 12-lead ECG signals to 3-lead VCG signals (Vx, Vy, Vz). Then VCG signals were converted into 2D RP images with no-normalization, min-max normalization, and z-scores normalization, respectively. These 2D RP maps were used to build 3DRP images, which were considered as the input data of the 3D neural network for training. As shown in Figure 7, it can be demonstrated apparent pattern differences between the VCG-based 3DRP images. The 3D networks learned feature maps embedded within these RP plots and between the leads, which contain arrhythmia type-dependent signatures, thus facilitating disease classification. The five-fold cross-validation was introduced in the training and validation processing, with the default parameters of Adam optimizer, a learning rate of 0.001, and a batch size of 64.

#### Experimental results

**FIGURE 8:**
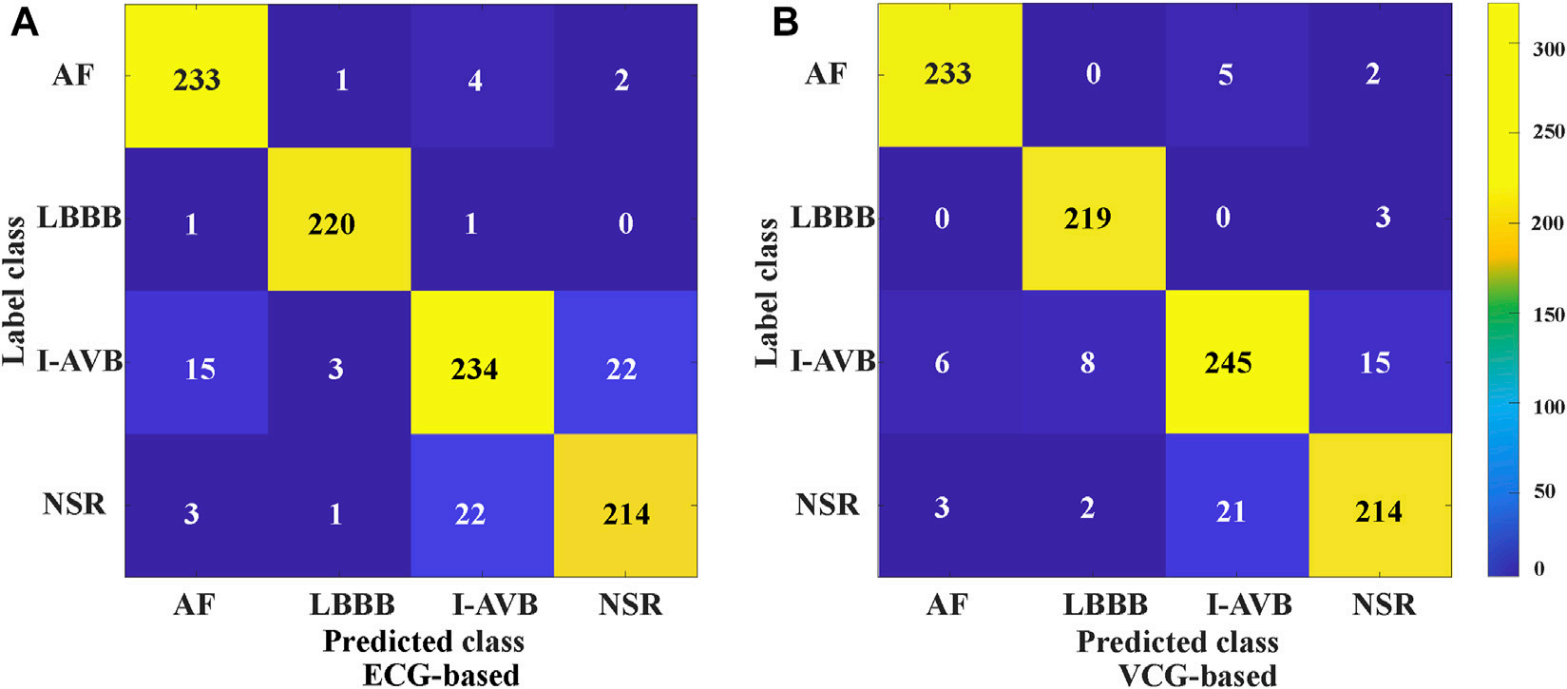
The confusion matrix of CA classification based on 3DRP ECG-based, and VCG-based.

The classification results of ECG-based and VCG-based 3DRP experiments are presented in Table 2. In this table, the method with z-score normalization achieved an Avg F1 score of 0.9254 for the ECG-based experiment and 0.9350 for the VCG-based experiment, outperforming other schemes. As shown in Table 3, the ECG-based experiment with z-score normalization obtained 0.9246 of the average Precision and 0.9269 of the average Recall. Besides, the highest F1-score was obtained for LBBB (0.9843), followed by AF (0.9472). In the VCG-based experiment with z-score normalization, the proposed method achieved the avg F1 score of 0.9350, the average Precision of 0.9344, and the average Recall of 0.9358. Besides, the highest F1 score was obtained for LBBB (0.9712), followed by AF (0.9668). Figure 8 is the arrhythmia classification confusion matrix of these two methods with z-score normalization. It outlines the data number of predicted and true labels. Note that there is a relatively small error between AF and LBBB, implying that the proposed method better predicts AF and LBBB.

**TABLE 2:** Classification performance based on ECG and VCG 3DRP methods with No/Min-max/Z-score normalization datasets.

Experiments	RP normalization	Avg F1-score	Classification of types of F1 score			
			NSR	AF	LBBB	I-AVB
ECG-based	No	0.9228	0.8847	0.9565	0.9775	0.8723
	Min-max	0.9247	0.8986	0.9407	0.9795	0.8799
	Z-score	0.9254	0.8954	0.9472	0.9843	0.8748
VCG-based	No	0.9301	0.9049	0.9610	0.9736	0.8810
	Min-max	0.9262	0.8946	0.9560	0.9692	0.8849
	Z-score	0.9350	0.9030	0.9668	0.9712	0.8991

**TABLE 3:** Classification Precision/Recall/F1-score of experiments.

Experiments	CA types	Precision	Recall	F1 score
ECG-based	NSR	0.8992	0.8917	0.8954
	AF	0.9246	0.9708	0.9472
	LBBB	0.9778	0.9910	0.9843
	I-AVB	0.8966	0.8540	0.8748
	Avg	0.9246	0.9269	0.9254
VCG-based	NSR	0.9145	0.8917	0.9030
	AF	0.9628	0.9708	0.9668
	LBBB	0.9563	0.9865	0.9712
	I-AVB	0.9041	0.8942	0.8991
	Avg	0.9344	0.9358	0.9350

#### Comparison of ECG-based and VCG-based 3DRP methods

This section compared the ECG-based experiment with the VCG-based experiment, focusing on network training and classification performance. Table 4 presents details of the training processing of each experiment. As indicated in the table, an equal number of trainable parameters were used in both methods. However, the training time of the 3-lead VCG-based method is 93 min, which is less than half of the 12-lead ECG method. The following columns show the fivefold cross-validation processing in terms of time and epochs used. Once the network is trained, it takes only 7ms and 16 ms for each prediction using the VCG-based and ECG-based methods, respectively. Table 3 compares the arrhythmia classification performances of these two methods. The optimal avg F1 score with VCG-based method is 0.9350, slightly better than the optimal ECG-based method (0.9254). The results highlight that the VCG-based method achieved a superior classification performance with less training time.

**TABLE 4:** Training information of the ECG-based and the VCG-based 3DRP methods.

Experiments	Trainable parameters	Training time	Five-fold validation				
			fold 1	fold 2	fold 3	fold 4	fold 5
ECG-based	27,038,708	262 Min	149 Min 56 Epochs	34 Min 11 Epochs	28 Min 11 Epochs	26 Min 11Epochs	25 Min 11Epochs
VCG-based	27,038,708	93 Min	33 Min 26 Epochs	21 Min 19 Epochs	13 Min 11 Epochs	13 Min 11Epochs	13 Min 11Epochs

### Comparison with different reference models

To study the reliability and effectiveness of the proposed method, we compared the performance of different reference models, including Resnet 50 (He et al., 2016), Inception-v3, and Inception-v4 (Szegedy et al., 2017). For a fair comparison, the same 3D VCG-based RP images were taken as the input of different models. The data were divided into training, validation, and testing sub-datasets using the same rule. Then, the same hyperparameters, including learning rate and batch size, were used to train and test the models separately. The average F1 score, Precision, and Recall of each class were calculated for comparison.

As illustrated in Table 5, the proposed method achieved the average F1 score of 0.9350, the average Precision of 0.9344, and the average Recall of 0.9358, which were all higher than those of other reference models. Moreover, it was shown that the proposed method outperformed the Resnet50, Inception V3, and Inception V4 in the F1 score of all classes. Interestingly, in the case of identifying the LBBB class, almost all the models achieved significantly higher F1 scores compared with other classes. Table 6 illustrates the computational costs of compared models. In five-fold cross-validation experiments, the training time of the proposed method is 93 min, which is less than that of other models except for the Inception V3 (71 min). And the number of trainable parameters of the proposed method is comparable with the Resnet 50 and the Inception V3, and less than the Inception V4 model.

**TABLE 5:** Comparison of different reference models for CA Classification.

Models	Classification of F1 score				Avg F1 score	Avg precision	Avg recall
	NSR	AF	LBBB	I-AVB			
RestNet50 (He et al., 2016)	0.8889	0.9339	0.9515	0.8791	0.9134	0.9116	0.9119
Inception V3 (Szegedy et al., 2017)	0.8683	0.9434	0.9556	0.8683	0.9089	0.9068	0.9068
Inception V4 (Szegedy et al., 2017)	0.8714	0.9263	0.9471	0.8355	0.8951	0.8920	0.8924
Proposed method	0.9030	0.9668	0.9712	0.8991	0.9350	0.9344	0.9358

**TABLE 6:** Comparison of the computational cost of the proposed 3D method *VS*. reference models.

Methods	Trainable parameters	Training time	Five-fold validation				
			fold 1	fold 2	fold 3	fold 4	fold 5
RestNet50 (He et al., 2016)	26,641,796	127 Min	55 Min 42 Epochs	27 Min 21 Epochs	16 Min 12 Epochs	14 Min 11 Epochs	15 Min 11 Epochs
Inception V3 (Szegedy et al., 2017)	21,831,844	71 Min	32 Min 32 Epochs	10 Min 11 Epochs	10 Min 11 Epochs	9 Min 11 Epochs	10 Min 11 Epochs
Inception V4 (Szegedy et al., 2017)	52,049,092	148 Min	72 Min 41 Epochs	17 Min 11 Epochs	25 Min 16 Epochs	17 Min 11 Epochs	17 Min 11 Epochs
**Proposed method**	27,038,708	93 Min	33 Min 26 Epochs	21 Min 19 Epochs	13 Min 11 Epochs	13 Min 11 Epochs	13 Min 11 Epochs

### Comparison of the proposed 3D method with recently published 1D and 2D methods

In this section, we compared the 3D RP VCG-based method with some recent CA classification studies, including the 1D raw ECG-based method (Hannun et al., 2019) and the 2D image-based method (Zhang et al., 2021), all are based on the same dataset PTB-XL. In the 1D case, the raw ECG time series were taken as the input to the model with 33 convolutional layers, and it outputs a prediction of one out of 4 possible rhythm classes every 256 input samples. In the 2D case, the 1D ECG data were converted into a set of 2DRP images fed into the 2D classification networks as the input, and the output was the prediction rhythm.

Table 7 and Table 8 show the comparison results, including the input, performance, and computing cost based on the five-fold cross-validation experiments. The 3D method obtained the highest average F1 score than the 1D and 2D approaches, with slightly longer training time than the 2D method and more complex networks than the 1D method. The proposed 3D method achieved better prediction performance for AF, LBBB, and I-AVB arrhythmia than the compared methods. At the same time, the 1D method achieved better performance for NSR, while the performance of the F1 score for the I-AVB (0.5833) is relatively low compared with the 2D approach (0.8503) and 3D method (0.8991), and the LBBB (0.8658) compared with the 2D approach (0.9267) and 3D method (0.9712), respectively.

**TABLE 7:** Comparison of performance of the proposed 3D method VS. 2D and 1D classification methods.

Methods	Input signals	Avg F1 score	Classification of subjects’ F1 score			
			NSR	AF	LBBB	I-AVB
1D (Hannun et al., 2019)	1D raw ECG	0.8483	0.9812	0.9627	0.8658	0.5833
2D (Zhang et al., 2021)	2D images	0.9015	0.8917	0.9365	0.9276	0.8503
Proposed method	3D images	0.9350	0.9030	0.9668	0.9712	0.8991

**TABLE 8:** Comparison of the computational costs of the proposed 3D method VS. 2D and 1D classification methods.

Methods	Trainable parameters	Training time	Five-fold validation				
			fold 1	fold 2	fold 3	fold 4	fold 5
1D (Hannun et al., 2019)	10,466,148	107 Min	36 Min 20 Epochs	16 Min 9 Epochs	16 Min 9 Epochs	21 Min 12Epochs	18 Min 10Epochs
2D (Zhang et al., 2021)	29,141,450	79 Min	46 Min 56 Epochs	9 Min 12 Epochs	8 Min 11 Epochs	8 Min 11 Epochs	8 Min 11Epochs
**Proposed method**	27,038,708	93 Min	33 Min 26 Epochs	21 Min 19 Epochs	13 Min 11 Epochs	13 Min 11Epochs	13 Min 11Epochs

### Testing the generalization of the proposed 3D method

In this section, we evaluated the generalization of the proposed approach by studying two more ECG datasets of the PhysioNet/Computing in Cardiology Challenge 2020. The detailed information of these two datasets is listed in Table 9. The data source CPSC (Liu et al., 2018) is the public training dataset from the China Physiological Signal Challenge (CPSC 2018). Georgia is a 12-lead ECG Challenge Database, Emory University, Atlanta, Georgia, United States, representing a large (Alday et al., 2021) population from the South-eastern United States.

**TABLE 9:** Generalization ability of the proposed method for CA classification on extra datasets.

Database	Mean duration	Number of subjects			
		NSR	AF	LBBB	I-AVB
CPSC	16.2s	918	1000	567	1422
Georgia	10.0s	1000	1054	438	1284

Database	Avg F1 score	Classification of subjects F1 score			
		NSR	AF	LBBB	I-AVB
CPSC	0.9412	0.9474	0.9497	0.9296	0.9381
Georgia	0.8881	0.9260	0.8723	0.8590	0.8950

In this experiment, raw ECG datasets were pre-processed and transformed into 3 lead VCG signals with the z-score normalization. As shown in Table 9, the proposed method achieved an average F1 score of 0.9412 on CPSC and 0.8881 on Georgia. The F1 score of each classification in CPSC is higher than in Georgia. The best prediction was obtained with an AF of 0.9497 on CPSC. For these two datasets, the proposed 3DRP method can effectively predict the AF, I-AVB, LBBB, and NSR. These testing results indicate that the 3DRP method has a good generalization for arrhythmia classification.

## Discussion

This work proposed a 3D method via extracting ECG signals’ dynamic, nonlinear recurrence features for deep learning-based arrhythmia classification. Instead of using 1D ECG and 2D ECG-based images, the 3D RP maps were reconstructed from 12 leads ECG and 3 leads VCG and then fed into the 3D CNN model for neural network training, validation, and testing. The superiority and effectiveness of the proposed method are validated by various experiments.

### The advantage of using the 3D method for CA classification

In 1D temporal ECG signals, dynamic nonlinear features and space-time characteristics are not directly observable. In our previous work (Zhang et al., 2021), the 2DRP method has demonstrated that recurrence plots help identify the nonlinear dynamic recurrent features hidden in the 1D ECG signal for better arrhythmia classification. We explore the feature differences between arrhythmia types from a novel 3D perspective, beyond the standard 1D ECG time series-based approach and the 2D images-based method. In this work, we compared the proposed 3D method with recently published studies based on the 1D raw ECG and 2D ECG-based images for CA classification in terms of the F1 score. The 3DRP method outperformed both the 1D method and the 2DRP approach considering both avg F1 score and the prediction for each type of arrhythmia (see Table 7). The avg F1-score is 0.9350 for the 3DRP method, significantly better than 0.8483 for the 1D method and 0.9015 for the 2D approach. The 3DRP method better characterizes the dynamic cardiac system in spatial/lead and temporal domains by exploiting higher-dimensional image information. They effectively identify the latent features of each arrhythmia type in the training processing. This working mechanism has effectively boosted the arrhythmia prediction performance.

### The use of VCG-based 3DRP plots for deep learning-based CA classification

As mentioned in *Vectocardiography Section*, VCG possesses several advantages over the standard ECG in representing spatiotemporal information of cardiac electrical activities (Meyers et al., 2020). Also, the 3 lead VCG based 3DRP image dataset is much smaller than the 12 lead ECG-based one. Our experiment (see Table 4) shows that the VCG-based 3DRP method achieved optimal performance with an average F1-score of 0.9350 over that of 0.9254 in ECG-based 3D method with less training time (93 min) than the ECG-based (262 min). In addition, the confusion matrix in Figure 8 illustrates that the VCG-based method can accurately classify AF and LBBB. Further investigation is required to study arrhythmia-specific prediction/classification.

Two extra ECG datasets of the PhysioNet/Computing in Cardiology Challenge 2020 were adopted to study the generalization of the proposed method. It achieved an average F1 score of 0.8881 on Georgia, and 0.9412 on CPSC, respectively. The results demonstrated that the 3D method has excellent generalization ability. In addition, the comparison among several neural networks is shown in Table 5 and Table 6; the proposed 3D Inception ResNet model offers better solutions with comparable computational cost over others, as measured by major assessment indicators.

### Computational cost

The 3D image-based learning scheme implemented in this work may lead to a concern of computational cost. The 3D model has fewer trainable parameters than 2D Inception-ResNet V2 models, as it practically improves the model structure and decreases the depth of layers. On the other hand, the 3D model has more trainable parameters than the 1D network. As demonstrated in Table 8, based on 3DRP reconstructed with 3 leads VCG, the five-fold cross-validation training time is 93 min, which is longer than the 2DRP-based method (79 min), but less than the 1D method (107 min). Thus, the computational cost is comparable among these 1D, 2D, and 3D methods. Moreover, as shown in the result section, 3DRP-based solutions offered significantly improved average F1 score and visual interpretability and boosted the prediction of types of arrhythmias (see Table 7). In particular, the VCG-based 3DRP solution provides the best performance in balancing accuracy and efficiency, making it appealing for clinical aid diagnosis.

## Conclusion

In this work, a 3D recurrence plot-based method was proposed for arrhythmia classification, achieving promising prediction performance with an inter-patient scheme. Compared with lower-dimensional classification methods, the proposed approach allows the learning algorithm to detect richer, nonlinear spatial-time features for better arrhythmia discrimination. Our simulation study confirmed that the 3D method offers superior performance to 1D/2D solutions and has a comparable computational cost.

## Data Availability

The original contributions presented in the study are included in the article/Supplementary material, further inquiries can be directed to the corresponding authors.
